# *Clostridium tertium* in Necrotizing Fasciitis and Gangrene

**DOI:** 10.3201/eid0910.030287

**Published:** 2003-10

**Authors:** Pallab Ray, Anindita Das, Kundan Singh, Anil Bhansali, T.D. Yadav

**Affiliations:** * Postgraduate Institute of Medical Education and Research, Chandigarh, India

**To the Editor:** Bacterial species of the genus *Clostridium* are anaerobic or aerotolerant, gram-positive, endospore-forming bacilli found in the soil and gut of humans and other animals. These species cause botulism, tetanus, gas gangrene, antibiotic-associated diarrhea, pseudomembranous colitis, foodborne diarrhea, and necrotic enteritis in humans and infections in other animals. *Clostridium tertium*, a non–exotoxin-producing, aerotolerant species, is an uncommon human pathogen. First isolated by Henry from war wounds in 1917 (*1*), *C. tertium* was recognized as a human pathogen when cases of bacteremia were reported in 1963 (*2*). The organism has been implicated in bacteremia (*3*,*4*), meningitis (*5*), septic arthritis (*6*), enterocolitis (*7*), spontaneous bacterial peritonitis (*8*), posttraumatic brain abscess (*9*), and pneumonia (*4*). Miller and colleagues, in a recent review of 32 cases, highlighted the role of neutropenia, intestinal mucosal injury, and exposure to β-lactam antibiotics predisposing to *C. tertium* bacteremia (*3*). *C. tertium* as the sole pathogen causing necrotizing fasciitis and gangrene has not been reported. We report the first two cases of necrotizing fasciitis and gangrene caused by *C. tertium*.

A 58-year-old man was seen at the Postgraduate Institute of Medical Education and Research, Chandigarh, India, on August 17, 2001, with two nonhealing, punched-out ulcers near the right lateral malleolus. The ulcers were approximately 2 cm x 2.5 cm in size, with necrotic margins, purulent exudate, and a foul odor. On the second day, temperature of 39.4°C and gangrene of the right leg developed. The patient also had alcoholic liver disease and non-Hodgkin lymphoma, for which he had been receiving chemotherapy for last 6 months, and he had had a 6 months’ course of antitubercular combination therapy for pulmonary tuberculosis 2 years earlier.

Peripheral blood showed a leukocyte count of 10,000/mm^3^ with 80% neutrophils, 11% lymphocytes, and 9% monocytes. The fasting blood glucose level was 400 mg/dL with normal electrolytes and renal function test results. Liver function tests showed a serum glutamic oxalacetic transaminase level of 80 IU/L and a serum glutamic pyruvic transaminase level of 75 IU/L. Sputum microscopy showed no acid-fast bacilli. Microscopy of necrotic tissue showed gram-positive bacilli (1–1.5 μm x 5–6 μm) with frequent oval terminal spores. Aerobic blood culture was sterile. Insulin was given to control his blood glucose level. Antimicrobial therapy included intravenous metronidazole, vancomycin, and imipenem. Skin and subcutaneous tissue debridement and fasciotomy were also undertaken.

A 40-year-old man was seen at the Postgraduate Institute on October 17, 2001, with multiple injuries of lower extremities and abdomen following a motor vehicle accident. After 24 h, the patient’s left leg and thigh turned gangrenous, and a high-grade fever (38.6°C) and an elevated leukocyte count of 14,000/mm^3^ (70% neutrophils, 20% lymphocytes, 10% monocytes) developed. Microscopy of necrotic tissue showed gram-positive rods (1 μm x 5 μm) with oval, terminal spores. Aerobic blood culture was sterile. Skin and subcutaneous tissue were extensively debrided. Antibiotic therapy with intravenous penicillin, metronidazole, and amikacin was instituted.

Necrotic tissues from both cases were cultured on Columbia sheep blood agar plates incubated aerobically and anaerobically (ANOXOMAT system, MART Microbiology BV, Lictenvoorde, the Netherlands) at 37°C for 24 h and 48 h, respectively. Overnight aerobic culture grew small gray colonies (<1 mm in diameter) of non–spore-forming gram-variable bacilli (1 μm x 5 μm), which, on subculture anaerobically grew gram-positive rods with oval, terminal spores. Anaerobic culture directly from specimen yielded similar spore-forming, gram-positive bacilli. The isolates were presumptively identified as *Clostridium* species by colony characteristics, Gram-stain morphology, and negative catalase test results; they were confirmed as *C. tertium* based on aerotolerance; shape and location of endospores; fermentation of glucose, lactose, maltose, and sucrose; nitrate reduction; and absence of proteolysis. Both isolates of *C. tertium* were susceptible in vitro to penicillin, ampicillin, vancomycin, and metronidazole.

*C. tertium* has been traditionally considered nonpathogenic. The organism was earlier isolated along with pathogens such as *C. perfringens*, *C. septicum,* and *C. sordellii* from war wounds and cases of gangrene (*1*). *C. tertium* is being increasingly reported as a human pathogen (*3*–*9*), and the strongest association has been with septicemia in patients with neutropenia and hematologic malignancies (*3*,*4*). Predisposing factors for *C. tertium* bacteremia include intestinal mucosal injury, neutropenia, β-lactam antibiotics (third-generation cephalosporins), cytotoxic chemotherapy, and severe liver disease, as reviewed by Miller and co-workers (*3*). Necrotizing fasciitis and gas gangrene caused by *C. tertium* as the sole pathogen have not been reported, although Miller reported necrotizing fasciitis in a patient with acute lymphocytic leukemia with *C. tertium* and *C. septicum* isolated from blood (*3*). The importance of isolation of *C. tertium*, particularly in polymicrobial cultures, is not well-established. In our report, the first patient suffered from alcoholic liver disease, had very high blood glucose levels, and was on cytotoxic chemotherapy for previous 6 months. The risk for intestinal injury is high in severe liver disease and cytotoxic chemotherapy. Intestinal mucosal compromise may potentiate translocation of *C. tertium* to systemic circulation and metastatic foci. The second patient had no predisposing medical history before the present episode that might have resulted in acquisition of *C. tertium* from the soil. Both patients had pyrexia, necrotizing fasciitis, and gangrene of a lower limb with *C. tertium* as the sole bacterial isolate. Neither patient had neutropenia when they were first seen. This contrasts with earlier reports of *C. tertium* infections (predominantly bacteremia), which usually occurred in patients with preexisting neutropenia (*3*). Both patients improved with penicillin or vancomycin and metronidazole, and both isolates were susceptible to these three antibiotics in vitro. Therefore, we consider both isolates to be clinically important. The pathogenesis of infection caused by *C. tertium* is not well understood, since the organism does not produce exotoxins. No evidence exists to correlate oxygen sensitivity with bacterial enzyme production and pathogenicity in aerotolerant clostridia. Our report adds to the list of recently emerging diseases caused by *C. tertium*. The growing acceptance of this organism as a human pathogen will lead to better delineation and understanding of its pathogenic potential.
